# Variation and evolution analysis of SARS-CoV-2 using self-game sequence optimization

**DOI:** 10.3389/fmicb.2024.1485748

**Published:** 2024-11-11

**Authors:** Ziyu Liu, Yi Shen, Yunliang Jiang, Hancan Zhu, Hailong Hu, Yanlei Kang, Ming Chen, Zhong Li

**Affiliations:** ^1^School of Information Engineering, Huzhou University, Huzhou, Zhejiang, China; ^2^College of Life Sciences, Zhejiang University, Hangzhou, Zhejiang, China; ^3^School of Computer Science and Technology, Zhejiang Normal University, Jinhua, Zhejiang, China; ^4^School of Mathematics, Physics and Information, Shaoxing University, Shaoxing, Zhejiang, China

**Keywords:** deep learning, SARS-CoV-2, evolution analysis, self-game sequence optimization, DARSEP model

## Abstract

**Introduction:**

The evolution of SARS-CoV-2 has precipitated the emergence of new mutant strains, some exhibiting enhanced transmissibility and immune evasion capabilities, thus escalating the infection risk and diminishing vaccine efficacy. Given the continuous impact of SARS-CoV-2 mutations on global public health, the economy, and society, a profound comprehension of potential variations is crucial to effectively mitigate the impact of viral evolution. Yet, this task still faces considerable challenges.

**Methods:**

This study introduces DARSEP, a method based on Deep learning Associates with Reinforcement learning for SARS-CoV-2 Evolution Prediction, combined with self-game sequence optimization and RetNet-based model.

**Results:**

DARSEP accurately predicts evolutionary sequences and investigates the virus’s evolutionary trajectory. It filters spike protein sequences with optimal fitness values from an extensive mutation space, selectively identifies those with a higher likelihood of evading immune detection, and devises a superior evolutionary analysis model for SARS-CoV-2 spike protein sequences. Comprehensive downstream task evaluations corroborate the model’s efficacy in predicting potential mutation sites, elucidating SARS-CoV-2’s evolutionary direction, and analyzing the development trends of Omicron variant strains through semantic changes.

**Conclusion:**

Overall, DARSEP enriches our understanding of the dynamic evolution of SARS-CoV-2 and provides robust support for addressing present and future epidemic challenges.

## Introduction

SARS-CoV-2 has spread more rapidly and extensively than initially anticipated since its detection in late 2019 (Ferretti et al., 2020; Worobey et al., 2022; Koelle et al., 2022). By January 2020, the World Health Organization (WHO) had declared this epidemic as a global public health emergency (Sohrabi et al., 2020). Until February 2023, the virus has caused approximately 600 million confirmed cases and over 6.5 million deaths in more than 200 countries, continuing to pose a significant threat to global health (Hus et al., 2023). Despite comprehensive prevention and control measures, SARS-CoV-2 continues to disseminate globally. Its remarkable mutability significantly hinders efforts to control the outbreak. Since the pandemic’s beginning, several highly transmissible SARS-CoV-2 variants, such as Alpha, Beta, Gamma, and Delta, have emerged (Pan et al., 2021; Singh et al., 2022). The Omicron variant, first identified in South Africa in November 2021, quickly became the dominant strain worldwide, characterized by its high transmissibility and ability to evade immunity (El-Shabasy et al., 2022). In some areas, Omicron’s infection rates have shown near-exponential growth, prompting the WHO to classify its global risk level as ‘very high’ (Cai et al., 2022; Khandia et al., 2022). Authorities and scientific communities are urgently evaluating the impact of existing containment and vaccination strategies and adapting approaches to address Omicron’s spread, as well as other variants (Petersen et al., 2022).

Regarding the evolution of the SARS-CoV-2, scientists consistently monitor and analyze emerging variants to assess their effects on vaccine efficacy and transmission dynamics (Ramesh et al., 2021). Given the Omicron and other variants’ high transmissibility and potential for immune evasion, researchers are actively exploring their genetic characteristics and impact on immunity. Current research on SARS-CoV-2 evolution falls into two main categories: studies based on biological experimental data and those using computational biology techniques. The former focuses on analyzing experimental data to explore the virus’s biological features, propagation mechanisms, and effects on host cells. The latter employs neural networks, machine learning, and other computational methods to predict mutation trends, diffusion pathways, and drug resistance, rapidly identifying new virus subtypes. This approach boasts high reproducibility and accurately reflects biological phenomena. For instance, researchers have used experimental data to study viral genomes and predict evolutionary paths (Wei et al., 2020; Ma et al., 2023; Zhao et al., 2023; Cao et al., 2023). They have explored the dissemination of mutant strains by examining changes in structure and receptor-binding properties (Harvey et al., 2021; Wrobel et al., 2022; Markov et al., 2023). Moreover, they have evaluated various models’ predictive power regarding viral evolution and identified different mutant strains, offering insights into potential evolutionary directions (Roemer et al., 2023). However, these studies require substantial experimental resources and long consuming time for analysis, presenting challenges for extensive analysis of viral antigenic proteins.

Consequently, machine learning-based methods are increasingly applied to predict and analyze evolutionary and mutational patterns in SARS-CoV-2. These models simulate viral-host interactions, predict crucial mutation sites’ effects on transmissibility, assess variants’ impact on binding affinity and immune evasion, forecast high-risk mutant strains, and track virus evolution across regions (Obermeyer et al., 2022; Taft et al., 2022; Makowski et al., 2022). Innovative approaches model the viral assembly process and potential mutation combinations, aiding in predicting future strains (Hajihosseinlou et al., 2023; Xu et al., 2023; Bakkas et al., 2023). Additionally, researchers have developed various deep learning models to predict SARS-CoV-2 mutations’ effects on immune evasion and infectivity. For example, Hie et al. and Singh Bist et al. used natural language processing to predict immune-evading mutations and identify key mutant sequences (Hie et al., 2021; Singh Bist et al., 2023). Chen et al. and Zvyagin et al. created models to forecast the binding affinity and antibody escape, monitoring SARS-CoV-2 variations (Chen et al., 2023; Zvyagin et al., 2022). Zhou et al. and Vaswani et al. combined the phylogenetic analysis and the Transformer framework to predict sequence mutations (Zhou et al., 2023), while Wang et al. introduced the UniBind framework to predict variations’ impact on protein interactions (Wang G. et al., 2023). Han W. et al. (2023) combined deep learning with genetic algorithms to predict antigen evolution. These methods enhance viral evolution monitoring and inform vaccine and therapeutic strategy development.

On the other hand, current machine learning and deep learning-based approaches for predicting SARS-CoV-2 evolution have several limitations. A notable issue is the prevalent reliance on traditional genetic algorithms to screen evolutionary sequences of SARS-CoV-2 (Whitley, 1994), which struggle to navigate the extensive array of viral variations due to their suboptimal performance in complex, high-dimensional spaces. This limitation could hinder the accurate identification of complex viral mutation patterns. Moreover, existing deep learning models encounter challenges in effectively modeling long viral sequences, affecting their predictive accuracy. Processing lengthy sequences demands significant computational resources without the guaranteed accuracy, potentially limiting the feasibility of training deep learning models on large datasets. Additionally, viral sequences contain detailed information on long-term dependencies and intricate structures, which current deep learning models may not capture accurately.

To address these challenges, we introduce a novel method called DARSEP (Deep learning Associates with Reinforcement learning for SARS-CoV-2 Evolution Prediction) for predicting and analyzing SARS-CoV-2 evolution. DARSEP involves constructing a model for SARS-CoV-2 spike proteins through a synergy of self-game sequence optimization and the RetNet framework (Sun et al., 2023). It utilizes the reinforcement learning in a self-playing model to explore the extensive mutation landscape of viral proteins and delineate the evolutionary path of SARS-CoV-2, focusing on optimizing the spike protein’s receptor-binding domain (RBD) to identify potential mutations. The spike protein sequences of SARS-CoV-2 are then modeled using the pre-trained protein language model ESM2 with the RetNet framework. The sequences refined through reinforcement learning are then subject to the downstream analysis, including semantic clustering, mutation site prediction, and missense mutation analysis, etc., aiming to elucidate the virus’s evolutionary dynamics. Experimental validations demonstrate DARSEP’s superiority over existing methods.

## Materials and methods

**FIGURE 1:**
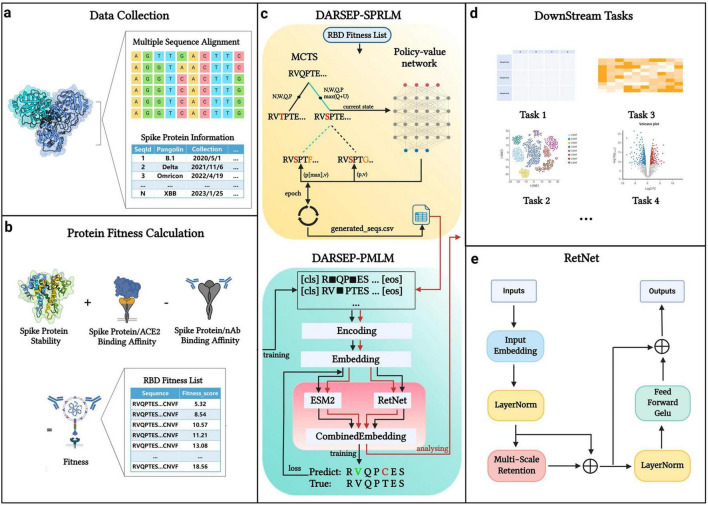
DARSEP model architecture. **(a)** Data collection and processing segment divides spike protein information into sequence data and additional data categories. **(b)** Fitness calculation for each sequence, which involves assessing folding free energy, receptor binding domain affinity, and antibody binding capability. **(c)** DARSEP-SPRLM is based on self-game reinforcement learning for optimizing sequences, incorporating Monte Carlo Tree Search and a policy-value network, to derive optimized sequences with their fitness values. **(d)** Downstream task analysis employs the trained DARSEP-PMLM model to evaluate optimized sequences through visualization and verification. **(e)** Network architecture diagram of the RetNet. Created in BioRender. L, Z. (2023) BioRender.com/q23y981.

The core architecture of our DARSEP model is illustrated in Figure 1. Initially, we collect SARS-CoV-2 sequence data from relevant databases for analysis (Figure 1a). After obtaining the viral sequences, we calculate their fitness values (Figure 1b) and use these data to train the spike protein reinforcement learning model, DARSEP-SPRLM, for sequence optimization (Figure 1c). Concurrently, we train the protein masked learning model, DARSEP-PMLM, using the same sequences (Figure 1c). Ultimately, we input both the original and optimized sequences into DARSEP-PMLM for the downstream task analysis (Figure 1d).

### Dataset

The primary data sources for our methodology are GISAID^1^ and NCBI Virus^2^ (Kalia et al., 2021; Brister et al., 2015). We obtained the amino acid sequences of SARS-CoV-2 spike proteins, dating from December 2019 to October 2023, along with sampling time, geographical information, and other pertinent data, predominantly from GISAID. The choice to focus on the SARS-CoV-2 spike protein, especially its receptor-binding domain (RBD), is due to the fact that this region, as the key to the viral invasion and the core of immune response, is not only directly related to the viral transmissibility, pathogenicity, and immune escape mechanism, but also has a decisive impact on vaccine development, antibody therapy, and diagnostic technology. The Pango lineage data for the virus is mainly sourced from the NCBI Virus database. For GISAID sequences that we are unable to obtain genealogical information directly from NCBI, we conduct the phylogenetic analysis based on the sequences themselves using public tools and software packages (e.g., Nextstrain, etc.) to reconstruct the approximate genealogical relationships of these sequences to ensure data integrity.

Upon collecting the spike protein sequences, in order to balance the inclusiveness of natural variability with data quality, and to ensure that the analysis focuses on a range of sequences that maintain critical structural and functional integrity, we filter out sequences in the length range of 1265–1273. We also exclude sequences containing more than 10 ‘X’ characters, representing unknown amino acids, and remove any duplicates. Following filtration, the sequences are subjected to multiple sequence alignment with MEGA11,^3^ and the region corresponding to the receptor-binding domain (RBD) is extracted. Finally, sequences from 2019 to 2021 are allocated as the training set, while those from 2022 to 2023 are used as the test set.

### Fitness calculation

The immunity developed through infection, vaccination, or passive immunization (via neutralizing antibodies) enhances the population’s resistance to SARS-CoV-2, thus increasing the selective pressure on the virus (Williams and Burgers, 2021). This pressure drives the virus to evolve, creating new variants that can evade the immune response and survive. To evaluate a virus’s ability to circumvent the immune system, we define “fitness” as its capacity to persist in the host, encompassing three critical attributes: protein stability, ACE2 binding affinity, and antibody binding affinity, described as follows:

(i) Protein stability

Protein stability is quantified by folding free energy, denoted as △*G*. Folding free energy (Honig and Yang, 1995), a pivotal concept in thermodynamics, characterizes a system’s stability and phase transition properties under varying external conditions. It encapsulates information about the system’s energy, entropy, and volume (Friston, 2010). Analyzing folding free energy enables understanding of phase transition principles, including changes in energy and entropy during this process. A mutation at any site in the protein sequence alters the folding free energy, represented as △△*G*. Assuming the wild type sequence’s folding free energy at each site is zero, the entire wild type sequence also has a folding free energy of zero. If a mutation alters the folding free energy at the *i*-th position in a sequence with length *L*, denoted as △△G*_i_*, the total change in folding free energy for the sequence, denoted as φ_E_, can be calculated as Equation 1:


(1) $$\varphi_{\text{E}}=\sum_{i=0}^{L}△\,△\,G_{i}$$

We use the PoPMuSiC (Dehouck et al., 2009) to determine the change in folding free energy due to mutations in the spike protein. This algorithm leverages the protein’s 3D structure and statistical mean-force potentials, converting them into free energies via the inverse Boltzmann law. When applying PoPMuSiC, we first retrieve the complete spike protein’s template structure, 6VYB (Ahmed et al., 2020), from Protein Data Bank (PDB). We then calculate the change in folding free energy for each site mutation across all amino acids. When φ_*E*_ > 0, the protein sequence moves towards stability, and when φ_*E*_ < 0, the protein sequence moves towards instability.

(ii) Affinity for ACE2 binding

We utilize the BeAtMuSiC (Dehouck et al., 2013) to evaluate the effect of individual genetic mutations on ACE2 binding affinity. This algorithm predicts the impact of potential genetic mutations on the binding strength to the ACE2 receptor. For this analysis, the 6M0J RBD/ACE2 complex structural template serves as the input (Veeramachaneni et al., 2021). Mirroring the methodology for calculating changes in folding free energy, we assume the ACE2 binding affinity of the wild-type sequence to be zero. We define $\Delta \,\Delta \,{\text{G}}_{i}^{\mathrm{ACE2}}$ as the change in binding affinity due to a mutation at the position *i* in an amino acid sequence with length *L*. The overall binding affinity of the entire amino acid sequence to ACE2 is denoted as Equation 2:


(2) $$\varphi_{\mathrm{ACE2}}=\sum_{i=0}^{L}\Delta \,\Delta \,G_{i}^{A\,C\,E\,2}$$

When φ_*ACE2*_ > 0, the protein sequence has an increased ability to bind to ACE2, and when φ_*ACE2*_ < 0, it indicates the reduced ACE2 binding affinity.

(iii) Antibody binding capability

We also employ the BeAtMuSiC algorithm to assess the change in binding capacity for 73 antibodies resulting from each potential single-site mutation in the spike protein. Antibody data are obtained from CoV-AbDab (Raybould et al., 2021), and by taking into account factors such as the broad neutralizing ability of antibodies, progress in clinical studies, frequency of citations in the literature, and scientific impact, we finally screen to obtain data with 73 antibodies, details of which can be found in the Supplementary file 3. We assume the baseline antibody binding capacity of the original sequence is zero. For an amino acid sequence of length *L*, the binding capacity of the *i-*th location changed to the *j-*th antibody is denoted as $\Delta \,\Delta \,G_{i}^{n\,A\,b,j}$. The average change in antibody binding capacity at each site, $\bar{\Delta \,\Delta \,{\text{G}}_{i}^{\text{nAb}}}$, represents the average of 73 alterations at each site, which is calculated as Equations 3, 4:


(3) $$\bar{\Delta \,\Delta \,G_{i}^{n\,A\,b}}=\frac{1}{73}\,\sum_{j=1}^{73}\Delta \,\Delta \,G_{i}^{n\,A\,b,j}$$

(4) $$\varphi_{n\,A\,b}=\sum_{i=0}^{L}\bar{\Delta \,\Delta \,G_{i}^{n\,A\,b}}$$

where φ_nAb_ denotes the alteration in the overall capacity of the sequence to adhere to the antibody. When φ_nAb_ > 0, the protein sequence becomes more capable of binding to the antibody, and when φ_nAb_ < 0, the protein sequence becomes less capable of binding to the antibody.

In summary, φ_*E*_ and φ_*ACE2*_ are positively correlated with the fitness of the spike protein sequence, and φ_nAb_ is negatively correlated with the fitness of the protein sequence, so the method of calculating the fitness φ of the spike protein sequence is the sum of φ_*E*_ and φ_*ACE2*_ with the difference of φ_nAb_. We treat three attributes with the same degree of importance, so the fitness of the viral protein sequence is calculated as Equation 5:


(5) $$\varphi =\varphi_{E}+\varphi_{A\,C\,E\,2}-\varphi_{\text{nAb}}$$

In the formulas for calculating fitness, we use a simplified approach that assigns equal weights to the three main factors that influence virus fitness. This simplification is intended to provide a balanced starting point for analysis in the absence of a priori information on specific viral environments. In the absence of clear evidence as to which factor dominates in all cases, assigning equal weights is a reasonable and practical approach. Furthermore, by normalizing the three factors, we ensure that they are on the same order of magnitude, thereby simplifying the model and ensuring that when assessing the various properties of the virus, it allows these properties to be compared on a fair scale. However, this approach ignores the complex interactions that may exist between factors in real-world situations. For example, in environments with low immune pressure, viruses may rely more on increasing their receptor binding efficiency to enhance infectivity, whereas in environments with high immune pressure, optimization of immune escape mechanisms may be more necessary to maintain their survival and spread. Future research should consider how to quantify the actual relative importance of these factors and adjust the weights in the model on a case-by-case basis to more accurately reflect changes in viral fitness in the real world.

### DARSEP-SPRLM for sequence optimization

Our sequence optimization approach, utilizing the AlphaZero reinforcement learning network (Silver et al., 2018), distinguishes itself from the genetic algorithm-based MLAEP method (Han W. et al., 2023). Reinforcement learning, in particular self-game mechanisms, mimics biological evolution to enable self-optimization of intelligences in strategy-critical domains (e.g., chess competition and protein engineering), where deep neural networks are fused by AlphaZero-inspired algorithms with Monte Carlo tree search to efficiently explore the huge design space and optimize sequence selection to reach the best functional properties. Reinforcement learning demonstrates unique advantages in sequence optimization because it can provide a solution that outperforms traditional machine/deep learning methods through dynamic adaptive strategies, automatic balance exploration and exploitation, learning generalization strategies from a small number of samples, optimizing directly for the final objective and efficiently dealing with the complexity of sequence decision making. In this framework, amino acids are analogized to chess pieces, mutations to moves, and sequence design to playing chess. We employ Monte Carlo Tree Search (MCTS) and a policy-value neural network to guide the model in identifying spike proteins with enhanced adaptiveness and potential for immune escape (Wang Y. et al., 2023). The detailed algorithm can be viewed in Supplementary file 1.

### DARSEP-PMLM for downstream analysis

The DARSEP-PMLM model integrates the pre-trained ESM2 (Lin et al., 2023) with the RetNet (Sun et al., 2023). ESM2 leverages an autoregressive neural network to assimilate evolutionary principles, elucidating the interrelations among protein sequences, structures, and functions. This model is trained on an extensive protein sequence database, enabling it to generate hidden vectors that encapsulate each protein sequence’s structural and functional attributes. RetNet, an adaptation of the Transformer model, utilizes a multi-scale retention mechanism, which addresses the ‘impossible triangle’ challenge - simultaneous achievement of parallel training, cost-effective inference, and robust scaling performance - faced by conventional neural network architectures. Our evaluations indicate that the ESM2 and RetNet combination surpasses the Transformer and other neural network models in prediction accuracy. Hence, we employ this dual approach for viral sequence modeling and analysis. The detailed algorithm can be viewed in Supplementary file 2.

### Evaluation metrics

In our experiment evaluation, the indicators for assessing the sequence optimization model DARSEP -SPRLM primarily include analyzing the capability of the optimized sequences, post -training on the dataset from 2019 to 2021, to accurately identify mutation sites in the test set spanning 2022 to 2023. Furthermore, the evaluation encompasses the Pearson correlation coefficient and the Canonical Correlation Analysis (CCA) coefficient between the predicted and actual fitness values derived from the optimized sequences, which are calculated using Equations 6, 7:


(6) $$\text{r}=\frac{\sum \left(x_{i}-\bar{x}\right)\,\left(y_{i}-\bar{y}\right)}{\sqrt{\sum {\left(x_{i}-\bar{x}\right)}^{2}\,{\left(y_{i}-\bar{y}\right)}^{2}}}$$

(7) $$\rho =\frac{C\,o\,v\,\left(X,Y\right)}{\sqrt{V\,a\,r\,\left(X\right)}\,\sqrt{V\,a\,r\,\left(Y\right)}}$$

where *x*_*i*_ and *y_i_* are the variables in two datasets *X* and *Y*, $\bar{x}$ and $\bar{y}$ are corresponding average values in *X* and *Y*, *Var* and *Cov* denote the variance and covariance computation.

For the DARSEP-PMLM, evaluation metrics are obtained through the mask training on the 2019 to 2021 training set and subsequently measuring Accuracy [Accuracy formula = (TP+TN)/ (TP+TN+FP+FN)], Precision [Precision formula = TP/(TP+FP)], Recall [Recall formula = TP/(TP+FN)], and F1-Score [F1 formula = (2 × Precision × Recall)/(Precision+ Recall)] on the 2022 to 2023 test set. These metrics are derived from the counts of True Positives (TP), True Negatives (TN), False Positives (FP), and False Negatives (FN).

## Results

The experimental results for DARSEP, a SARS-CoV-2 evolutionary prediction model, are divided into two primary sections. The first section includes a self-play reinforcement learning model utilizing the AlphaZero algorithm (Silver et al., 2018). The agent’s function is enhanced by processing sequences of RBDs and their corresponding fitness labels through simulation accumulation and strategic iteration, continuously generating new RBD sequences indicative of the virus’s potential evolutionary paths. The second section introduces a novel model for predicting and analyzing SARS-CoV-2 evolution, integrating the pre-trained ESM2 with RetNet framework to examine the optimized spike protein sequences.

### Dataset

Our research concentrates on the RBD region of the SARS-CoV-2 spike protein. The dataset, sourced from GISAID and NCBI, includes spike protein sequences from the outbreak’s inception until 2021 as the training set, with sequences from 2022 to 2023 serving as the test set. To maintain consistency, we normalize the lengths of the viral sequences to 1273 by aligning them. Following the extraction of the RBD region and the elimination of duplicates, we compile a dataset of 23,633 unique sequences.

### Sequence optimization experiment

#### Calculation of fitness

**FIGURE 2:**
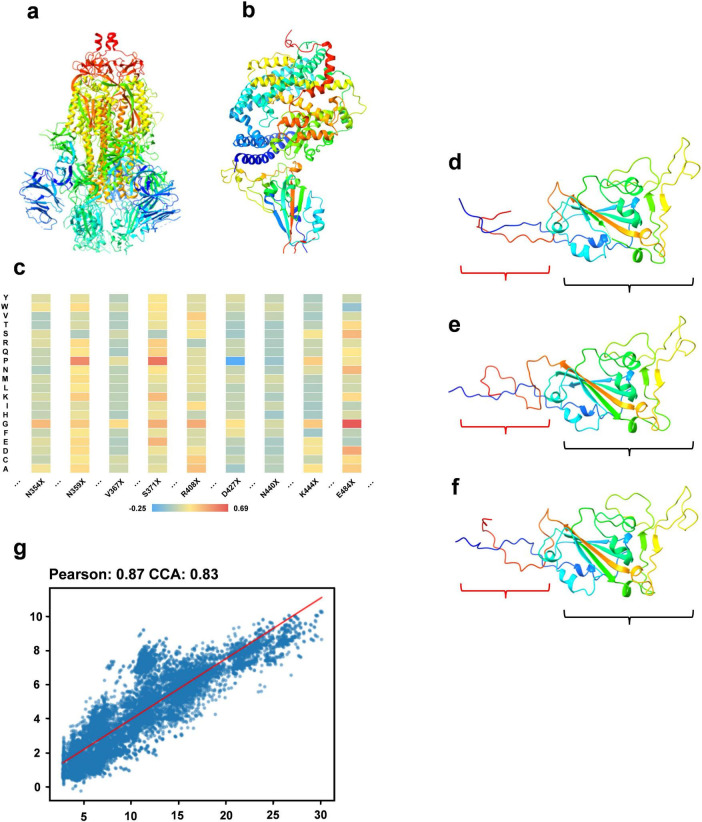
Sequence optimization experiment. **(a)** The structure of the SARS-CoV-2 spike ectodomain (6VYB). **(b)** The 3D structure of the RBD/ACE2 complex. **(c)** A heat map illustrating fitness variations resulting from mutations at specific RBD region sites to different amino acids. **(d)** The 3D structure of a sequence within the optimized RBD sequence set. **(e)** The 3D structure of the wild type sequence. **(f)** The 3D structure of the sequence most similar to the optimized sequence in the multiple sequence alignment. **(g)** A regression analysis showing the correlation between the predicted fitness of the optimized sequence from our model and the actual calculated fitness post optimization.

The constituents of fitness include stability (measured by folding free energy), ACE2 binding capacity, and antibody binding capacity. We utilize the PoPMuSiC, which incorporates the protein’s 3D structure and a statistical average force potential, to assess stability. Using only the SARS-CoV-2 spike ectodomain structure (6VYB, open state) as the input for the algorithm (Figure 2a), enabling the computation of energy changes (ΔΔG) for mutations at each site to different amino acids. For ACE2 binding capacity evaluation, the BeAtMuSiC algorithm is applied, similarly, solely using the 3D structure of the RBD/ACE2 complex (6M0J) as input (Figure 2b). This facilitates the estimation of potential energy changes due to mutations at specific sites. To assess mutations’ effects on antibody binding, we analyze their impact on 73 antibodies using BeAtMuSiC, calculating the average mutation-induced change for each antibody at each RBD site. Summing these averages provides an overall mutation impact at each RBD site. Aggregating these effects yields the sequence’s total fitness, with the wild type sequence assigned a fitness value of 0. Figure 2c depicts the fitness variations due to mutations at particular sites, displayed as a heat map.

#### Generation of optimized RBD sequences

We use RBD sequences from 2019 to 2021 as the training set, with associated fitness values as labels. Our DARSEP-SPRLM model conducts sequence optimization, resulting in a collection of optimized RBD sequences and their fitness values. Analysis of mutation sites reveals a significant number of mutations in these optimized sequences either appear in the 2022 to 2023 test set or are absent in the training set. Table 1 shows 7 of mutation sites predicted by the DARSEP-SPRLM model during sequence optimization, and it is noteworthy that these predicted mutations are subsequently observed in reality, confirming the effectiveness and potential of the model in anticipating emerging mutations. To validate the structural integrity of these sequences, We use AlphaFold2 (Jumper et al., 2021) for the exclusive purpose of predicting the three-dimensional structure of selected sequences and comparing them to wild-type sequences and similar sequences in multiple sequence comparisons. Comparisons, as depicted in Figures 2d–f, evaluate the correlation between the predicted and calculated fitness of optimized sequences by our model. This correlation is further substantiated in Figure 2g, showing a Pearson’s coefficient of 0.87 and a CCA coefficient of 0.83. The structural prediction results show that the optimized sequences are slightly structurally different from the wild type and similar variants (regions enclosed in red brackets in Figures 2d–f), but the main body maintains a high degree of structural similarity (regions enclosed in black brackets in Figures 2d–f), which implies that the optimization process preserves the core structural features of the sequences. The correlation coefficients underscore our model’s strong predictive ability, accurately estimating the fitness levels of optimized sequences. In summary, our optimization model proficiently generates new RBD sequences that closely resemble the original ones in structure and function, yet include numerous new mutation sites, providing valuable insights for subsequent experimental research.

**TABLE 1:** List and verification of some mutations

Site	Origin	Mutation	GISAID ID	Pango linage	Collection date	Location
330	P	V	EPI_ISL_17372618	XBB	2023-01-16	India
337	P	I	EPI_ISL_11755367	BA.2.3.11	2022-03-09	Thailand
352	A	E	EPI_ISL_17585762	BA.1	2022-09-14	Egypt
415	T	G	EPI_ISL_16394747	BA.2.10.1	2022-12-16	India
421	Y	Q	EPI_ISL_12714856	BA.1.1	2022-01-01	USA
431	G	Q	EPI_ISL_12589413	BA.2	2022-04-30	Spain
466	R	V	EPI_ISL_16553496	BQ.1.1	2023-01-03	USA

It is widely recognized that viral protein sequence variants, especially those conservative mutations, usually do not lead to significant structural changes in the protein. The current model effectively captures this relationship between viral mutations and structural stability, maintaining structural integrity while optimizing the viral sequence. Moreover, beneficial genetic variations become prevalent and spread throughout the population through natural selection (Nielsen et al., 2014). This model enhances our understanding of how organisms adapt to environmental changes by simulating a self-driven survival competition process. In virology, viruses can develop new adaptive mutations in response to the host immune system and drug interventions (Han A. X. et al., 2023). Our model can mimic the adaptive evolutionary process of viruses, predicting their evolutionary paths (Section 3.2). Additionally, there is a quantifiable link between a biomolecule’s function and structure (Lyon et al., 2021), and the fitness scoring system in this model offers an objective method to evaluate sequence-function relationships. Overall, biological evolution is guided by random mutations and natural selection, and our model can replicate this mutation selection process, generating new viral sequences with optimal fitness levels.

### DARSEP training and downstream task analysis

**FIGURE 3:**
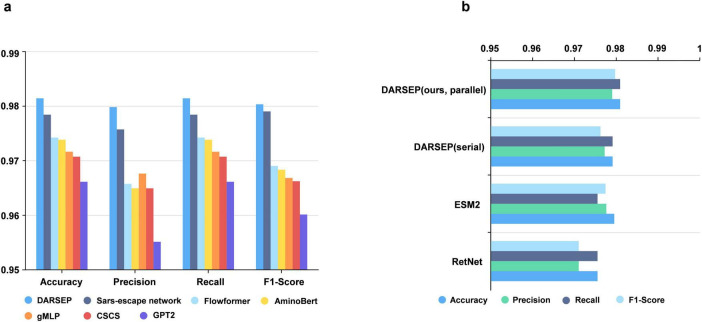
Performance analysis of the DARSEP model. **(a)** Comparison of the performance of different models for viral sequence mask training, including DARSEP, Sars-escape network, Flowformer, AminoBert, gMLP, CSCS, GTP2. **(b)** The ablation study for DARSEP is depicted, where ESM2 denotes the DARSEP model without RetNet, and RetNet indicates the DARSEP model sans ESM2.

Training of the DARSEP-PMLM model. DARSEP-PMLM integrates a pre-trained ESM2 protein language model with a Transformer-based RetNet, training on sequence masks from RBD sequences sourced from GISAID. This process enables the model to grasp both positional relationships and semantic syntactic patterns within sequences. A comparative analysis of DARSEP with various sequence modeling frameworks, such as Flowformer (Wu et al., 2022), Sars-escape network (Singh et al., 2022), AminoBert (Chowdhury et al., 2022), CSCS (Hie et al., 2021), gMLP (Liu et al., 2021) and GPT2 (Brown et al., 2020), based on Accuracy, Precision, Recall, and F1 Score, reveals that our model excels in all these metrics (Figure 3a), prompting its application in further downstream analyses.

### Ablation experiment

We conduct ablation studies to elucidate the contribution of each model within DARSEP, with separate training sessions for ESM2 and RetNet. ESM2’s independent training underscores its pivotal role in decoding protein language, affirming its substantial impact on the model’s predictive accuracy. Similarly, isolated training of RetNet emphasizes its importance in protein language modeling. Evaluation of RetNet’s standalone performance confirms its synergistic effect with ESM2, enhancing overall model efficacy. The best results are observed when ESM2 and RetNet are combined in the full DARSEP-PMLM model. The ablation study outcomes are illustrated in Figure 3b.

### Downstream task analysis - clustering analysis

**FIGURE 4:**
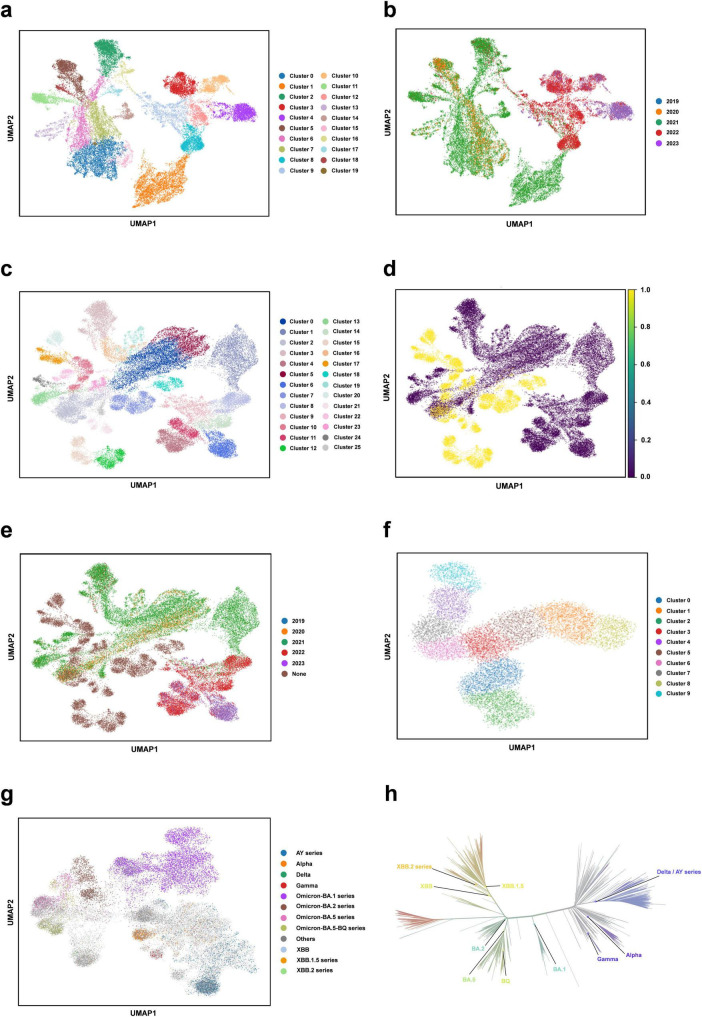
Clustering analysis of our protein language model. **(a)** Semantic embedding clustering plot for the training sequence dataset. **(b)** Semantic embedding clustering plot for the training sequence dataset, colored by year. **(c)** Semantic embedding clustering plot combining the training and optimized datasets. **(d)** Semantic embedding clustering plot for the training and optimized datasets, with training data in purple and optimized data in yellow. **(e)** Semantic embedding clustering plot for the training and optimized dataset, colored by year. **(f)** Semantic embedding clustering plot for the optimized sequence set. **(g)** Clustering plot colored for different variants with Pango lineages. **(h)** Phylogenetic tree of SARS-CoV-2 (from https://nextstrain.org/).

We perform the clustering analysis using the trained DARSEP-PMLM model. Initially, we compute the semantic embedding for the training and optimized sequence sets. We then employ the UMAP (McInnes et al., 2018), a nonlinear dimensionality reduction technique, for manifold learning and the visualization of these embeddings. Following this, we utilize Leiden’s method (Traag et al., 2019) for the graph-based clustering on three sets of sequences: training, optimized, and a combined set. For visualization, we assign distinct colors to represent the clustering outcomes, as illustrated in Figure 4. We did the following analysis:

(i) Leiden clustering effectively separates various classes of viral protein sequences into distinct groups within the training set, mirroring the divergence patterns of natural evolution. Each cluster signifies a notable evolutionary path. Despite originating from the same virus, the sequences in different clusters have undergone substantial evolutionary changes, leading to divergent evolutionary paths. When clusters are colored based on the year attribute, early-phase sequences of the virus appear more closely grouped. Over time, the sequences start to exhibit varied evolutionary trajectories, and the sequence characteristics evolve, highlighting a shift in the genetic linkages between older and newer strains. This finding offers critical insights for predicting new strain emergence and viral evolutionary trends.
(ii) Leiden clustering of the three sets shows that optimized sequences cluster with the training sequences, indicating that the new sequences retain considerable genetic resemblance to the original despite optimization, without showing significant genetic drift. Coloring the clusters by year reveals that the new sequences diverge markedly from the originals, forming distinct evolutionary branches.
(iii) We also color different variants with Pango lineages, and viral sequences belonging to the same lineage are all tightly clustered within the same region, which further confirm the genetic consistency among Pango lineages. Each cluster represents a unique evolutionary branch, and these branches show significant genetic differences over time, forming their own unique patterns. The clustering relationships between clusters, such as the clustering distances exhibited between Omicron-BA.2 and Omicron-BA.5-BQ, also reflect similar positional relationships in the phylogenetic tree shown in Figure 4h. This high degree of consistency suggests that our clustering method can effectively reflect the homology features during virus evolution and that the clustering results largely encompass populations that are taxonomically homologous to the phylogenetic tree of virus evolution.
(iv) We also considered the effect of geography on clustering. With the results obtained from Leiden clustering, we analyzed from a geographic perspective that geographic isolation and host range restriction are intertwined, contributing to the uniqueness of genetic variation in different host environments. As time evolves, these genetic differences accumulate and solidify, gradually forming specific evolutionary branches. Specifically, at long time scales, viral sequences exhibit diverse evolutionary pathways, and geographic isolation, as one of the key driving forces (Li et al., 2021), further accelerates sequence divergence in this process, resulting in the emergence of independent lineages with distinct genetic signatures. Therefore, it can be concluded that time course and geographic heterogeneity exhibit a strong correlation in Leiden clustering analyses and are key dimensions in understanding the dynamics of the genetic structure of virus populations. The geographic distribution of viral populations and their historical changes should be considered in future assessments of their genetic diversity. This is crucial for our understanding of viral evolutionary patterns and for the development of effective public health strategies. Overall, this clustering analysis validates our protein sequence optimization model’s effectiveness, demonstrating its capability to accurately interpret and analyze the semantic properties of viral sequences.

The primary sources of variation in viral genome sequences are: (1) site mutations, which lead to amino acid substitutions, altering the structure and function of viral proteins (Saha et al., 2020), and (2) genome recombination, resulting in the creation of chimeric viruses (Perez-Losada et al., 2015). These random mutations, coupled with the selective forces of natural selection, drive the targeted variation and adaptive evolution of viral genomes across different host environments. Additionally, geographical isolation and limited host range contribute to distinct genetic variations within various host contexts over time, culminating in unique evolutionary lineages (Pybus and Rambaut, 2009). When comparing newly identified virus strains with older ancestral ones, a degree of genetic similarity often persists, reflecting their evolutionary connections. The model introduced in this study offers an in-depth insight into aspects like randomness, natural selection pressures, and homology, enabling precise simulation of the mutation and evolutionary processes of viral genomes. Moreover, employing clustering and visualization analyses illustrate the temporal evolution of viral populations, which could assist in predicting future viral genome mutations and provide essential information for virus surveillance.

### Downstream task analysis - constructing evolutionary fields

**FIGURE 5:**
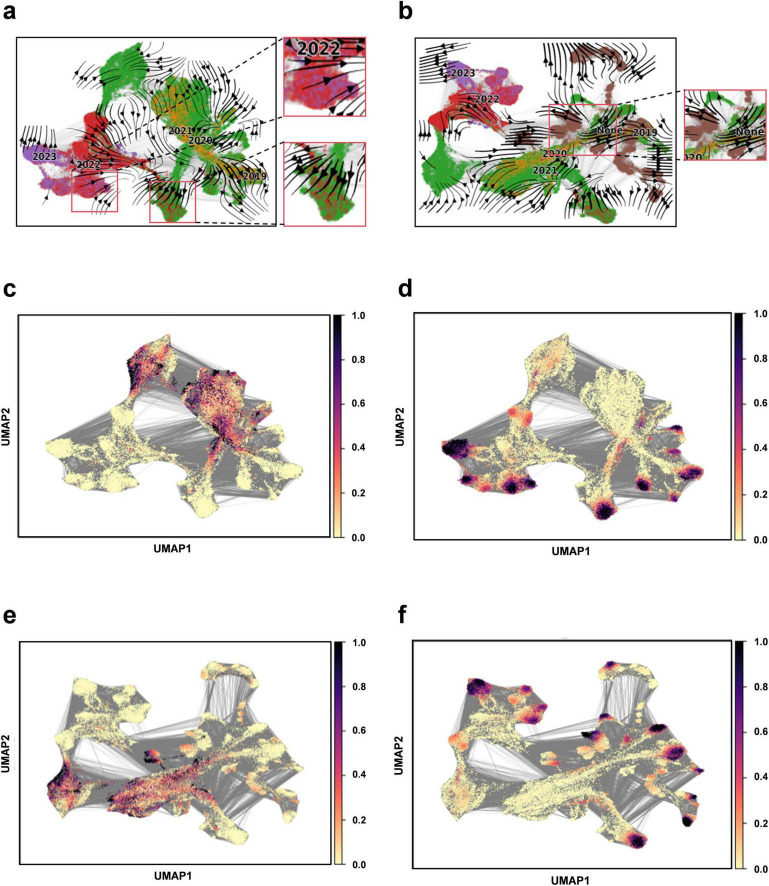
Construction of evolutionary vector fields. **(a)** Flow chart illustrating the evolution of RBD sequences from 2019 to 2023. **(b)** Flow chart illustrating the evolution of RBD sequences for the complete set (including 2019–2023 and optimized sequences). **(c)** Prediction plot for pseudotime at the beginning of sampling (2019–2023). **(d)** Predicting plot for pseudotime at the end of sampling (2019–2023). **(e)** Prediction plot for pseudotime at the beginning of sampling for the entire sequence set (2019–2023 and optimized sequences). **(f)** Prediction plot for pseudotime at the end of sampling for the entire sequence set (2019–2023 and optimized sequences).

We apply Evo-velocity analysis to create a dynamic evolutionary vector field for SARS-CoV-2 RBD sequences as part of our downstream task analysis (Hie et al., 2022). The Evo-velocity diagram (Figure 5) visually represents the RBD sequences from 2019 to 2023, including the optimized sequence set. An interesting observation emerges when we construct the temporal evolution field for this period: there is a strong correlation between the direction of viral evolution and time. Specifically, the viral evolutionary trajectories show a clear pattern over time. Additionally, we find a significant correlation between the sampling date and “pseudo time” (a metric to describe the evolution rate and direction of the virus), further substantiating the influence of time on viral evolution. Notably, the potential evolutionary direction of the overall sequence vector field appears to be aligning with our optimized sequences. This finding implies that the optimized sequences may represent the predominant route for the virus’s future evolution. Therefore, it is crucial to closely monitor these sequences and utilize them as primary indicators for understanding and forecasting viral evolutionary dynamics, offering enhanced insights for virus prevention and control.

Viral genome sequences experience continuous diversification due to random site mutations and the directional influence of natural selection pressures, illustrating the dynamic equilibrium between random mutation and natural selection, akin to random drift and the Ornstein-Uhlenbeck process (Nadeau et al., 2023). The “pseudotemporal” parameter, quantifying the rate and pattern of genetic changes in the viral population, exhibits a strong correlation with both geological factors and time, underscoring the temporal dependency and indicative nature of viral evolution (Gupta et al., 2022). In this model, the optimized sequences are oriented towards the likely direction of future evolution, ensuring retention of genetic information from original sequences while accurately representing the predominant trends in viral evolution. This alignment suggests a connection to the Markov stochastic process hypothesis, illustrating the commonality and shared lineage in viral evolution. To forecast new strain developments, it is crucial to establish time series data that can track the evolutionary dynamics of viral mutations over time. By amalgamating sequencing data from different time points, we can depict the temporal progression of viral evolution, offering valuable insights into viral population dynamics. Our findings indicate that the model effectively mirrors the temporal patterns of viral sequence changes and possesses the capability to predict future viral mutation trajectories.

### Downstream task analysis - prediction of missense variant effects

**FIGURE 6:**
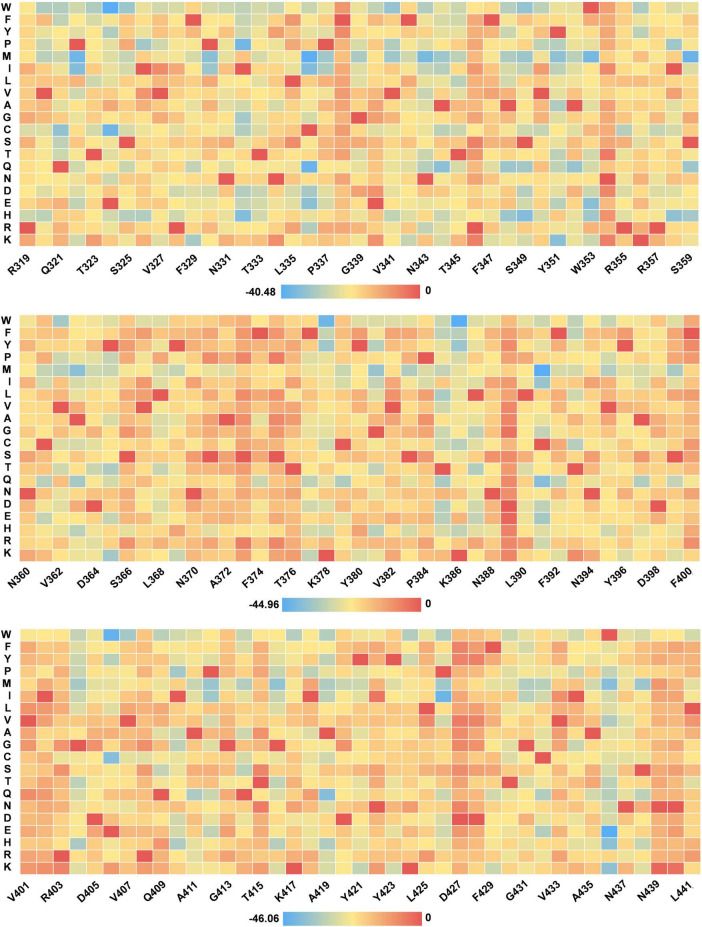
Heat map of missense mutation scores for selected RBD sequences.

We evaluate the effects of all possible missense variants within the RBD region using our trained protein language model, assessing the impact of these variants at every position. The effect score for each variant is calculated based on the log-likelihood ratio (LLR) between the variant and wild type (WT) residues. Figure 6 illustrates this, displaying a heatmap of variant effects at specific sites. A detailed account of all missense variant effects is available in the Supplementary file 4. It is important to note that a higher score indicates a more detrimental effect of the mutation, as observed with G339D in the Omicron variant series.

The LLR method applies the dN/dS ratio from molecular evolution to distinguish between neutral and functionally impactful human variations (Nadeau et al., 2023). Variants with elevated LLR scores may significantly disrupt the RBD structure. Alterations in key binding site residues can affect their chemistry, reducing binding affinity and impacting protein stability. Modifications in hydrogen bonding, hydrophobic interactions, charge, and other factors could lead to reduced RBD stability, increasing the propensity for aggregation and decreasing solubility (Rosace et al., 2023). Notably, the high- frequency mutations N501Y and E484A identified in our analysis show high LLR scores, validating the LLR score as a reliable metric for predicting the functional impact of variations.

### Downstream task analysis—epidemic strain analysis

**FIGURE 7:**
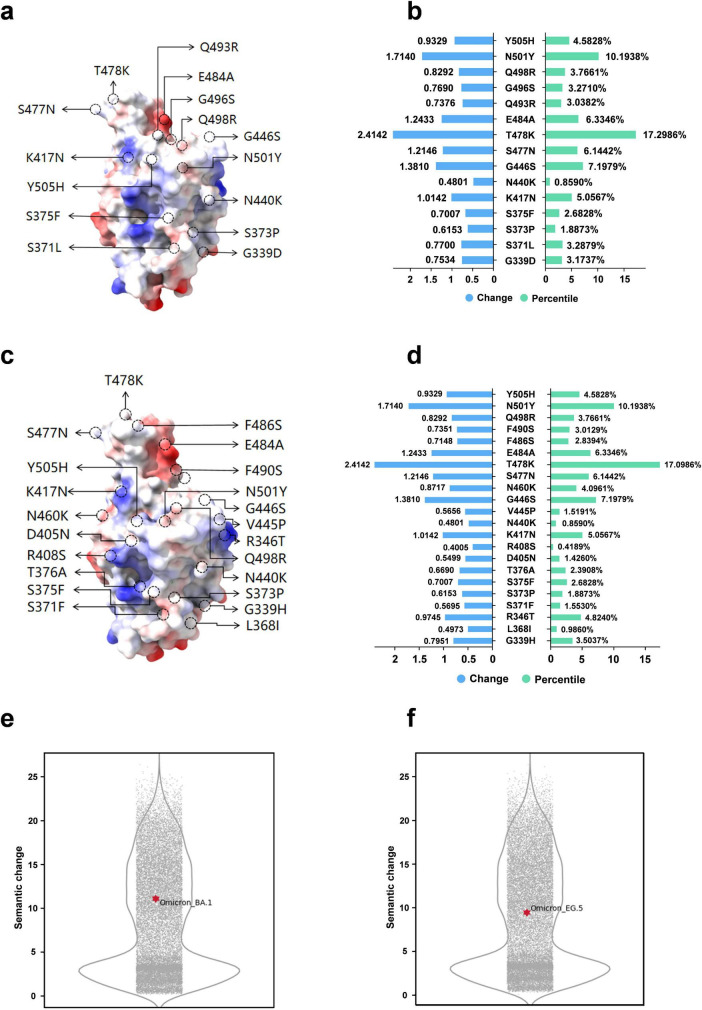
Semantic analysis of Omicron mutation strains. **(a)** Mutation sites in the RBD region of Omicron strain BA.1. **(b)** Semantic changes and percentage rankings at mutation sites in the RBD region of Omicron strain BA.1. **(c)** Mutation sites in the RBD region of Omicron strain EG.5. **(d)** Semantic changes and percentage ranking in the RBD region of Omicron strain EG.5. **(e)** Violin plot depicting semantic changes for the Omicron strain BA.1 (semantic change value: 10.36). **(f)** Violin plot depicting semantic changes for the Omicron strain EG.5 (semantic change value: 9.56).

We analyze two Omicron variant lineages: BA.1, noted for its heightened transmissibility, and EG.5, which is currently prevalent. We also delineate the structural mutation sites for these variants, illustrated in Figures 7a,c. Utilizing our model, we determine the percentile rankings for semantic changes at each mutation site (Figures 7b,d). Additionally, we create violin plots to depict the distribution of semantic changes for these two variants, highlighting their positions within the entire viral mutation landscape (Figures 7e,f). Semantic changes reflect the impact of amino acid substitutions on protein function, hinting at how mutations modify viral traits. Syntactic changes pertain to the physicochemical property alterations of the substituted amino acids, representing the mutation’s magnitude. Integrating both provides a comprehensive assessment of a mutation’s influence. Our analysis reveals that sites with substantial semantic shifts and high percentile rankings, like T478K found in Delta and Omicron strains, are associated with major epidemics. The T478K mutation is implicated in enhancing the spike protein’s interaction with its receptor, potentially aiding the immune evasion. The N501Y mutation, identified early, shows a marked increase in affinity for the human ACE2 receptor over the wild type, significantly boosting the virus’s infectivity and transmissibility (Liu et al., 2022). Quantitative comparison of semantic changes between the BA.1 and EG.5 strains, visualized through violin plots, indicates a reduction in semantic changes and associated risks from BA.1 to EG.5. Despite EG.5 becoming the dominant strain, a comprehensive WHO assessment in early August 2023 concluded that EG.5 does not pose a novel or escalated threat (Parums, 2023). This analysis of semantic and syntactic changes corroborates established virological insights and validates our method’s efficacy.

## Discussion

In this study, we introduce DARSEP, a novel method for the variation and evolution analysis of SARS-CoV-2. Initially, we utilize a self-play strategy to navigate the extensive mutation landscape, aiming to identify optimal evolutionary trajectories for viral proteins. Following this, we train a sophisticated protein language model combining ESM2 and RetNet, which surpasses other leading models. Using this model, we analyze the optimized sequences to gain deep insights into viral evolution patterns. Our methodology successfully predicts the evolution trajectory of the virus and identifies key mutation sites that could enhance viral adaptability and immune evasion. Importantly, our model can foresee potential mutations based on data from 2019 to 2021, facilitating early detection and the formulation of public health strategies. By utilizing all available sequence data to date, the model may identify viral sequences that are expected to appear in the future, exhibit high fitness, and possess significant potential for survival and transmission. These highly fitnessed sequences indicate potential evolutionary trajectories for viruses, particularly in response to immune selection pressures and other environmental constraints. Subsequent to the evaluation of these high-fitness sequences, a series of downstream tasks were executed alongside protein language model. Clustering identifies sequences that closely align with known sequences, indicating a degree of genetic similarity while also exhibiting new characteristics that may differentiate them as distinct evolutionary branches in the future. Moreover, by examining the semantic syntax of the novel sequences, one can pinpoint mutation locations that perform exceptionally in percentile rankings. These loci are likely to attract future scrutiny, as their existence suggests that the virus may have developed increased adaptive capabilities. Sequences with numerous highly scored mutation sites are more likely to dominate future epidemics. This series of studies enhances our comprehension of viral evolutionary processes and provide essential support for vaccine modification tactics and antiviral medication development. This approach will enable us to forecast viral evolutionary patterns and furnish public health policymakers with timely information to formulate more effective preventative and control strategies against potential future dangers.

The proposed method effectively delineates the evolutionary landscape of SARS-CoV-2, enhancing our understanding of its evolutionary dynamics and providing substantial scientific backing to tackle current and forthcoming epidemics. Nevertheless, our study has limitations that warrant further refinement. At present, our study has focused on specific regions of spike protein sequences and their single site mutational effects, and has not yet involved epistasis / mutation interactions or explored the effects of other mutation types such as deletions and insertions. And we use a fixed set of 73 antibodies as the basis for assessing SARS-CoV-2 fitness, a treatment that ignores dynamic changes in population immunity due to repeated infections, the application of emerging vaccines, and the discovery of new antibodies, which may lead to limitations in the accuracy of the model’s simulation in a rapidly evolving immune environment. We acknowledge that viral evolution is a complex process involving various mutation types. Future research needs to explore a wider range of mutation types and interactions, and construct models that incorporate dynamic immune responses to enhance the prediction accuracy and optimize the outbreak prevention and control strategy. Moreover, there is an opportunity to extend our model to encompass additional viral attributes, including host immune responses and virus-host cell interaction dynamics. It is also crucial to integrate insights from diverse fields such as data mining, biology, medicine, and public health to deepen our grasp of viral evolutionary processes and forecast the consequences of potential mutations.

## Data Availability

The original contributions presented in the study are included in the article/Supplementary material, further inquiries can be directed to the corresponding author.

## Footnotes

1
https://gisaid.org/

2
https://www.ncbi.nlm.nih.gov/labs/virus/vssi/

3
www.megasoftware.net

## References

[B1] Ahmed S. A. Abdelrheem D. A. El-Mageed H. R. A. Mohamed H. S. Rahman A. A. Elsayed K. N. M. (2020). Destabilizing the structural integrity of COVID-19 by caulerpin and its derivatives along with some antiviral drugs: An in silico approaches for a combination therapy. *Struct. Chem.* 31 2391–2412. 10.1007/s11224-020-01586-w 32837118 PMC7376526

[B2] Bakkas J. Hanine M. Chekry A. Gounane S. de la Torre Diez I. Lipari V. (2023). SARSMutOnto: An ontology for SARS-CoV-2 lineages and mutations. *Viruses* 15:505. 10.3390/v15020505 36851719 PMC9967353

[B3] Brister J. R. Ako-Adjei D. Bao Y. Blinkova O. (2015). NCBI viral genomes resource. *Nucleic Acids Res.* 43 D571–D577. 10.1093/nar/gku1207 25428358 PMC4383986

[B4] Brown T. B. Mann B. Ryder N. Subbiah M. Kaplan J. Dhariwal P. (2020). Language models are few-shot learners. *arXiv* [Preprint]. arXiv: 2005.14165.

[B5] Cai W. Yang Z. Liang J. Lin Z. Ma Y. Chen C. (2022). How fast and how well the Omicron epidemic was curtailed. A Guangzhou experience to share. *Front. Public Health* 10:979063. 10.3389/fpubh.2022.979063 36620243 PMC9812567

[B6] Cao Y. Jian F. Wang J. Yu Y. Song W. Yisimayi A. (2023). Imprinted SARS-CoV-2 humoral immunity induces convergent Omicron RBD evolution. *Nature* 614 521–529. 10.1038/s41586-022-05644-7 36535326 PMC9931576

[B7] Chen J. Nie Z. Wang Y. Wang K. Xu F. Hu Z. (2023). Running ahead of evolution—AI-based simulation for predicting future high-risk SARS-CoV-2 variants. *Int. J. High Perform.* 37 650–665. 10.1177/10943420231188077

[B8] Chowdhury R. Bouatta N. Biswas S. Floristean C. Kharkar A. Roy K. (2022). Single-sequence protein structure prediction using a language model and deep learning. *Nat. Biotechnol.* 40 1617–1623. 10.1038/s41587-022-01432-w 36192636 PMC10440047

[B9] Dehouck Y. Grosfils A. Folch B. Gilis D. Bogaerts P. Rooman M. (2009). Fast and accurate predictions of protein stability changes upon mutations using statistical potentials and neural networks: PoPMuSiC-2.0. *Bioinformatics* 25 2537–2543. 10.1093/bioinformatics/btp445 19654118

[B10] Dehouck Y. Kwasigroch J. M. Rooman M. Gilis D. (2013). BeAtMuSiC: Prediction of changes in protein-protein binding affinity on mutations. *Nucleic Acids Res.* 41 W333–W339. 10.1093/nar/gkt450 23723246 PMC3692068

[B11] El-Shabasy R. M. Nayel M. A. Taher M. M. Abdelmonem R. Shoueir K. R. Kenawy E. R. (2022). Three waves changes, new variant strains, and vaccination effect against COVID-19 pandemic. *Int. J. Biol. Macromol.* 204 161–168. 10.1016/j.ijbiomac.2022.01.118 35074332 PMC8782737

[B12] Ferretti L. Wymant C. Kendall M. Zhao L. Nurtay A. Abeler-Dorner L. (2020). Quantifying SARS-CoV-2 transmission suggests epidemic control with digital contact tracing. *Science* 368:abb6936. 10.1126/science.abb6936 32234805 PMC7164555

[B13] Friston K. (2010). The free-energy principle: A unified brain theory? *Nat. Rev. Neurosci .* 11 127–138. 10.1038/nrn2787 20068583

[B14] Gupta R. Cerletti D. Gut G. Oxenius A. Claassen M. (2022). Simulation-based inference of differentiation trajectories from RNA velocity fields. *Cell Rep. Methods* 2:100359. 10.1016/j.crmeth.2022.100359 36590685 PMC9795361

[B15] Hajihosseinlou M. Maghsoudi A. Ghezelbash R. (2023). A novel scheme for mapping of MVT-Type Pb–Zn Prospectivity: LightGBM, a highly efficient gradient boosting decision tree machine learning algorithm. *Nat. Resour. Res.* 32 2417–2438. 10.1007/s11053-023-10249-6

[B16] Han A. X. de Jong S. P. J. Russell C. A. (2023). Co-evolution of immunity and seasonal influenza viruses. *Nat. Rev. Microbiol.* 21 805–817. 10.1038/s41579-023-00945-8 37532870

[B17] Han W. Chen N. Xu X. Sahil A. Zhou J. Li Z. (2023). Predicting the antigenic evolution of SARS-COV-2 with deep learning. *Nat. Commun.* 14:3478. 10.1038/s41467-023-39199-6 37311849 PMC10261845

[B18] Harvey W. T. Carabelli A. M. Jackson B. Gupta R. K. Thomson E. C. Harrison E. M. (2021). SARS-CoV-2 variants, spike mutations and immune escape. *Nat. Rev. Microbiol.* 19 409–424. 10.1038/s41579-021-00573-0 34075212 PMC8167834

[B19] Hie B. L. Yang K. K. Kim P. S. (2022). Evolutionary velocity with protein language models predicts evolutionary dynamics of diverse proteins. *Cell Syst.* 13 274–285.e276. 10.1016/j.cels.2022.01.003 35120643

[B20] Hie B. Zhong E. D. Berger B. Bryson B. (2021). Learning the language of viral evolution and escape. *Science* 371 284–288. 10.1126/science.abd7331 33446556

[B21] Honig B. Yang A. S. (1995). Free energy balance in protein folding. *Adv. Protein. Chem.* 46 27–58. 10.1016/s0065-3233(08)60331-9 7771321

[B22] Hus I. Szymczyk A. Manko J. Drozd-Sokolowska J. (2023). COVID-19 in adult patients with hematological malignancies-lessons learned after three years of pandemic. *Biology (Basel)* 12:545. 10.3390/biology12040545 37106746 PMC10136203

[B23] Jumper J. Evans R. Pritzel A. Green T. Figurnov M. Ronneberger O. (2021). Highly accurate protein structure prediction with AlphaFold. *Nature* 596 583–589. 10.1038/s41586-021-03819-2 34265844 PMC8371605

[B24] Kalia K. Saberwal G. Sharma G. (2021). The lag in SARS-CoV-2 genome submissions to GISAID. *Nat. Biotechnol.* 39 1058–1060. 10.1038/s41587-021-01040-0 34376850

[B25] Khandia R. Singhal S. Alqahtani T. Kamal M. A. El-Shall N. A. Nainu F. (2022). Emergence of SARS-CoV-2 Omicron (B.1.1.529) variant, salient features, high global health concerns and strategies to counter it amid ongoing COVID-19 pandemic. *Environ. Res.* 209:112816. 10.1016/j.envres.2022.112816 35093310 PMC8798788

[B26] Koelle K. Martin M. A. Antia R. Lopman B. Dean N. E. (2022). The changing epidemiology of SARS-CoV-2. *Science* 375 1116–1121. 10.1126/science.abm4915 35271324 PMC9009722

[B27] Li J. Lai S. Gao G. F. Shi W. (2021). The emergence, genomic diversity and global spread of SARS-CoV-2. *Nature* 600 408–418. 10.1038/s41586-021-04188-6 34880490

[B28] Lin Z. Akin H. Rao R. Hie B. Zhu Z. Lu W. (2023). Evolutionary-scale prediction of atomic-level protein structure with a language model. *Science* 379 1123–1130. 10.1126/science.ade2574 36927031

[B29] Liu H. Dai Z. So D. R. Le Q. V. (2021). Pay attention to MLPs. *arXiv* [Preprint]. arXiv: 2105.08050.

[B30] Liu Y. Liu J. Plante K. S. Plante J. A. Xie X. Zhang X. (2022). The N501Y spike substitution enhances SARS-CoV-2 infection and transmission. *Nature* 602 294–299. 10.1038/s41586-021-04245-0 34818667 PMC8900207

[B31] Lyon A. S. Peeples W. B. Rosen M. K. (2021). A framework for understanding the functions of biomolecular condensates across scales. *Nat. Rev. Mol. Cell. Biol.* 22 215–235. 10.1038/s41580-020-00303-z 33169001 PMC8574987

[B32] Ma W. Fu H. Jian F. Cao Y. Li M. (2023). Immune evasion and ACE2 binding affinity contribute to SARS-CoV-2 evolution. *Nat. Ecol. Evol.* 7 1457–1466. 10.1038/s41559-023-02123-8 37443189

[B33] Makowski E. K. Schardt J. S. Smith M. D. Tessier P. M. (2022). Mutational analysis of SARS-CoV-2 variants of concern reveals key tradeoffs between receptor affinity and antibody escape. *PLoS Comput. Biol.* 18:e1010160. 10.1371/journal.pcbi.1010160 35639784 PMC9223403

[B34] Markov P. V. Ghafari M. Beer M. Lythgoe K. Simmonds P. Stilianakis N. I. (2023). The evolution of SARS-CoV-2. *Nat. Rev. Microbiol.* 21 361–379. 10.1038/s41579-023-00878-2 37020110

[B35] McInnes L. Healy J. Saul N. Großberger L. (2018). UMAP: Uniform manifold approximation and projection. *J. Open Source Softw.* 3:61. 10.21105/joss.00861

[B36] Nadeau S. A. Vaughan T. G. Beckmann C. Topolsky I. Chen C. Hodcroft E. (2023). Swiss public health measures associated with reduced SARS-CoV-2 transmission using genome data. *Sci. Transl. Med.* 15:eabn7979. 10.1126/scitranslmed.abn7979 36346321 PMC9765449

[B37] Nielsen K. M. Bohn T. Townsend J. P. (2014). Detecting rare gene transfer events in bacterial populations. *Front. Microbiol.* 4:415. 10.3389/fmicb.2013.00415 24432015 PMC3882822

[B38] Obermeyer F. Jankowiak M. Barkas N. Schaffner S. F. Pyle J. D. Yurkovetskiy L. (2022). Analysis of 6.4 million SARS-CoV-2 genomes identifies mutations associated with fitness. *Science* 376 1327–1332. 10.1126/science.abm1208 35608456 PMC9161372

[B39] Pan T. Hu Z. Hu F. Zhang Y. Liu B. Ke C. (2021). Significantly reduced abilities to cross-neutralize SARS-CoV-2 variants by sera from convalescent COVID-19 patients infected by Delta or early strains. *Cell Mol. Immunol.* 18 2560–2562. 10.1038/s41423-021-00776-8 34635805 PMC8503867

[B40] Parums D. V. (2023). Editorial: A rapid global increase in COVID-19 is due to the emergence of the EG.5 (Eris) subvariant of omicron SARS-CoV-2. *Med. Sci. Monit.* 29:e942244. 10.12659/MSM.942244 37654205 PMC10478578

[B41] Perez-Losada M. Arenas M. Galan J. C. Palero F. Gonzalez-Candelas F. (2015). Recombination in viruses: Mechanisms, methods of study, and evolutionary consequences. *Infect. Genet. Evol.* 30 296–307. 10.1016/j.meegid.2014.12.022 25541518 PMC7106159

[B42] Petersen E. Ntoumi F. Hui D. S. Abubakar A. Kramer L. D. Obiero C. (2022). Emergence of new SARS-CoV-2 variant of concern Omicron (B.1.1.529) - highlights Africa’s research capabilities, but exposes major knowledge gaps, inequities of vaccine distribution, inadequacies in global COVID-19 response and control efforts. *Int. J. Infect. Dis.* 114 268–272. 10.1016/j.ijid.2021.11.040 34863925 PMC8634699

[B43] Pybus O. G. Rambaut A. (2009). Evolutionary analysis of the dynamics of viral infectious disease. *Nat. Rev. Genet.* 10 540–550. 10.1038/nrg2583 19564871 PMC7097015

[B44] Ramesh S. Govindarajulu M. Parise R. S. Neel L. Shankar T. Patel S. (2021). Emerging SARS-CoV-2 variants: A review of its mutations, its implications and vaccine efficacy. *Vaccines (Basel)* 9:1195. 10.3390/vaccines9101195 34696303 PMC8537675

[B45] Raybould M. I. J. Kovaltsuk A. Marks C. Deane C. M. (2021). CoV-AbDab: The coronavirus antibody database. *Bioinformatics* 37 734–735. 10.1093/bioinformatics/btaa739 32805021 PMC7558925

[B46] Roemer C. Sheward D. J. Hisner R. Gueli F. Sakaguchi H. Frohberg N. (2023). SARS-CoV-2 evolution in the Omicron era. *Nat. Microbiol.* 8 1952–1959. 10.1038/s41564-023-01504-w 37845314

[B47] Rosace A. Bennett A. Oeller M. Mortensen M. M. Sakhnini L. Lorenzen N. (2023). Automated optimisation of solubility and conformational stability of antibodies and proteins. *Nat. Commun.* 14:1937. 10.1038/s41467-023-37668-6 37024501 PMC10079162

[B48] Saha P. Banerjee A. K. Tripathi P. P. Srivastava A. K. Ray U. (2020). A virus that has gone viral: Amino acid mutation in S protein of Indian isolate of Coronavirus COVID-19 might impact receptor binding, and thus, infectivity. *Biosci. Rep.* 40:1312. 10.1042/BSR20201312 32378705 PMC7225408

[B49] Silver D. Hubert T. Schrittwieser J. Antonoglou I. Lai M. Guez A. (2018). A general reinforcement learning algorithm that masters chess, Shogi, and Go through self-play. *Science* 362 1140–1144. 10.1126/science.aar6404 30523106

[B50] Singh Bist P. Tayara H. To Chong K. (2023). Sars-escape network for escape prediction of SARS-COV-2. *Brief. Bioinform.* 24:bbad140. 10.1093/bib/bbad140 37039673

[B51] Singh H. Dahiya N. Yadav M. Sehrawat N. (2022). Emergence of SARS-CoV-2 new variants and their clinical significance. *Can. J. Infect. Dis. Med. Microbiol.* 2022:7336309. 10.1155/2022/7336309 35669528 PMC9167142

[B52] Sohrabi C. Alsafi Z. O’Neill N. Khan M. Kerwan A. Al-Jabir A. (2020). World Health Organization declares global emergency: A review of the 2019 novel coronavirus (COVID-19). *Int. J. Surg.* 76 71–76. 10.1016/j.ijsu.2020.02.034 32112977 PMC7105032

[B53] Sun Y. Dong L. Huang S. Ma S. Xia Y. Xue J. (2023). Retentive network: A successor to transformer for large language models. *arXiv* [Preprint]. 10.48550/arXiv.2307.08621

[B54] Taft J. M. Weber C. R. Gao B. Ehling R. A. Han J. Frei L. (2022). Deep mutational learning predicts ACE2 binding and antibody escape to combinatorial mutations in the SARS-CoV-2 receptor-binding domain. *Cell* 400:e4014. 10.1016/j.cell.2022.08.024 36150393 PMC9428596

[B55] Traag V. A. Waltman L. van Eck N. J. (2019). From Louvain to Leiden: Guaranteeing well-connected communities. *Sci. Rep.* 9:5233. 10.1038/s41598-019-41695-z 30914743 PMC6435756

[B56] Veeramachaneni G. K. Thunuguntla V. Bobbillapati J. Bondili J. S. (2021). Structural and simulation analysis of hotspot residues interactions of SARS-CoV 2 with human ACE2 receptor. *J. Biomol. Struct. Dyn.* 39 4015–4025. 10.1080/07391102.2020.1773318 32448098 PMC7284149

[B57] Wang G. Liu X. Wang K. Gao Y. Li G. Baptista-Hon D. T. (2023). Deep-learning-enabled protein-protein interaction analysis for prediction of SARS-CoV-2 infectivity and variant evolution. *Nat. Med.* 29 2007–2018. 10.1038/s41591-023-02483-5 37524952

[B58] Wang Y. Tang H. Huang L. Pan L. Yang L. Yang H. (2023). Self-play reinforcement learning guides protein engineering. *Nat. Mach. Intell.* 5 845–860. 10.1038/s42256-023-00691-9

[B59] Wei X. Li X. Cui J. (2020). Evolutionary perspectives on novel coronaviruses identified in pneumonia cases in China. *Natl. Sci. Rev.* 7 239–242. 10.1093/nsr/nwaa009 32288962 PMC7107983

[B60] Whitley D. (1994). A genetic algorithm tutorial. *Stat. Comput.* 4:5354. 10.1007/BF00175354

[B61] Williams T. C. Burgers W. A. (2021). SARS-CoV-2 evolution and vaccines: Cause for concern? *Lancet Respir. Med.* 9 333–335. 10.1016/S2213-2600(21)00075-8 33524316 PMC8009632

[B62] Worobey M. Levy J. I. Malpica Serrano L. Crits-Christoph A. Pekar J. E. Goldstein S. A. (2022). The Huanan seafood wholesale market in wuhan was the early epicenter of the COVID-19 pandemic. *Science* 377 951–959. 10.1126/science.abp8715 35881010 PMC9348750

[B63] Wrobel A. G. Benton D. J. Roustan C. Borg A. Hussain S. Martin S. R. (2022). Evolution of the SARS-CoV-2 spike protein in the human host. *Nat. Commun.* 13:1178. 10.1038/s41467-022-28768-w 35246509 PMC8897445

[B64] Wu H. Wu J. Xu J. Wang J. Long M. (2022). Flowformer: Linearizing transformers with conservation flows. *arXiv* [Preprint]. arXiv: 2202.06258.

[B65] Xu Z. Wei D. Zeng Q. Zhang H. Sun Y. Demongeot J. (2023). More or less deadly? A mathematical model that predicts SARS-CoV-2 evolutionary direction. *Comput. Biol. Med.* 153:106510. 10.1016/j.compbiomed.2022.106510 36630829 PMC9816089

[B66] Zhao Y. Ni W. Liang S. Dong L. Xiang M. Cai Z. (2023). Vaccination with S(pan), an antigen guided by SARS-CoV-2 S protein evolution, protects against challenge with viral variants in mice. *Sci. Transl. Med.* 15:eabo3332. 10.1126/scitranslmed.abo3332 36599007

[B67] Zhou B. Zhou H. Zhang X. Xu X. Chai Y. Zheng Z. (2023). TEMPO: A transformer-based mutation prediction framework for SARS-CoV-2 evolution. *Comput. Biol. Med.* 152:106264. 10.1016/j.compbiomed.2022.106264 36535209 PMC9747230

[B68] Zvyagin M. Brace A. Hippe K. Deng Y. Zhang B. Bohorquez C. O. (2022). GenSLMs: Genome-scale language models reveal SARS-CoV-2 evolutionary dynamics. *bioRxiv* [Preprint]. 10.1101/2022.10.10.511571 36451881 PMC9709791

