# Supplementary material for: Variation and evolution analysis of SARS-CoV-2 using self-game sequence optimization
Source: Front Microbiol. 2024 Nov 11;15:1485748. doi: 10.3389/fmicb.2024.1485748 (PMC11586374; doi:10.3389/fmicb.2024.1485748)
Supplement: Supplementary file 1 [file Table_1.docx]

DARSEP-SPRLM Algorithm

The environment state *S* in reinforcement learning consists of a binary matrix with $20\times L$, where 20 represents the different types of amino acids and $L$ is the sequence length. This matrix can be conceived as a chess board, where a value of 1 indicates a “move”, that is, a single-site mutation at a given position, and 0 indicates that there is no mutation at that position. Action *A* denotes a precise amino acid modification at a sequence position, altering the state *S.* Each move's status *t* and its associated reward are indicated by variables $s_{t}$ and $r_{t}$, respectively.

The input for our model comprises amino acid sequences along with their corresponding fitness values, with each sequence represented using one-hot encoding. The optimization process unfolds in three distinct stages: the self-game learning phase, the training neural network phase, and the assessment network phase. During the self-game phase, moves are determined using the MCTS. Each node within a tree possesses three attributes: N(s,a),P(s,a) and Q(s,a), where N represents the number of visits to the node, P denotes the probability or strategy of selecting the next move, and Q reflects the current node's value. Initially, the UCB (Upper Confidence Bound) approach (Wang et al., 2023) is applied to select the node with the highest value. The UCB value for a node is calculated using the formula:

$$U(s,a)=cP(s,a)\frac{\sqrt{\sum N(s,b)}}{1+N(s,a)}$$

where *c* is a constant, *b* denotes the action taken at the parent node, and $N(s,b)$ represents the number of visits to the parent node. Subsequently, the action *a* with the highest score is determined by maximizing the sum of $U(s,a)$ and $Q(s,a)$:

$$a=argmax(Q(s,a)+U(s,a))$$

We continue the search process as described above until reaching a leaf node *S ^L^*, include this node in the search tree and set the parameters to their starting values

$$N(s^{L},a)=0,Q(s^{L},a)=0, P(s^{L},a)=0$$

We employ the strategy-value neural network model $f_{\theta}$ to predict the probability distribution and state values $p^{L},v^{L}$ of the optional actions corresponding to the leaf node$s^{L}$ to obtain $P(s,a)$. Subsequently, we retrace our steps sequentially back to the initial node, and simultaneously update the value and visiting count of each node on the backtracking path

$$(p^{L},v^{L})=f_{\theta}(s^{L})$$

$$N(s,a)=N(s,a)+1$$

$$Q(s,a)=\frac{v^{L}+(N(s,a)-1)Q(s,a)}{N(s,a)}$$

The model's loss function consists of three parts: the first is the mean square error loss function, which assesses the difference between the predicted and actual win/loss outcomes. The second is the cross-entropy loss function, measuring the discrepancy between the neural network's output strategy and the MCTS-generated strategy. The third is the L2 regularization term, which helps prevent overfitting by penalizing large weights

$$\mathcal{L=}{(r-v^{L})}^{2}-\pi^{T}log p+\alpha\left\| \theta\right\|^{2}$$

$$\pi(a|s)=\frac{{N(s,a)}^{\frac{1}{\tau}}}{\sum_{b} {N(s,b)}^{\frac{1}{\tau}}}$$

where the reward value for each move is denoted by $r$, the strategy produced by MCTS is represented by $\pi$, the adjustable temperature parameter is denoted by $\tau$, and the regularisation parameter for $\theta$ is represented by $\alpha$. In essence, this model inputs a set of amino acid sequences along with their fitness values. Post-training, the model yields an optimized set of sequences with their predicted fitness values.

# Reference

Wang, Y., Tang, H., Huang, L., Pan, L., Yang, L., Yang, H., et al. (2023). Self-play reinforcement learning guides protein engineering. *Nat. Mach. Intell.* 5(8)**,** 845-860. doi: 10.1038/s42256-023-00691-9.
