# Supplementary material for: Variation and evolution analysis of SARS-CoV-2 using self-game sequence optimization
Source: Front Microbiol. 2024 Nov 11;15:1485748. doi: 10.3389/fmicb.2024.1485748 (PMC11586374; doi:10.3389/fmicb.2024.1485748)
Supplement: Supplementary file 2 [file Table_2.docx]

DARSEP-PMLM Algorithm

Inputs are spike protein sequences and each sequence with length *L* is denoted as $x\mathbf{=(}x_{1}\mathbf{,}x_{2}, ... ,x_{L}\mathbf{)}$, where $x_{i}\in\{All amino acids\}$. A portion of amino acids is masked out by a random mask to obtain the masked sequence $x_{\mathrm{mask}}=\mathbf{(}x_{1}\mathbf{,}x_{2}, [mask] ,... , [mask] ,x_{L}\mathbf{)}$, and then the $x_{\mathrm{mask}}$ is encoded into a numeric sequence $x_{\mathrm{encode}}=\mathbf{(}x_{1}\mathbf{,}x_{2}, ... ,x_{L}\mathbf{)}$ by the encoder of ESM2. All these sequences in the training set are uniformly encoded into a two-dimensional matrix dataset $X$ denoted by the numbers, and the masked amino acids form the labelled set $Y$.

Initially, an embedding calculation is required to transform the amino acids for compatibility with the model's input:

$$X_{Embedding}=Embedding(X)$$

Upon acquiring the computationally embedded dataset X_Embedding, we input it into the pre-trained protein language model ESM2 to derive the structure, function, and additional pertinent features of the sequence, which is characterized as follows:

$$X_{feat\_1}=ESM2(X_{Embedding})$$

Subsequently, we utilize the RetNet to facilitate the modeling of the spike protein sequences. This architecture maintains a similarity to the Transformer, incorporating a LayerNorm layer succeeded by the Muti-Scale Retention (MSR) layer, as illustrated in Fig. 1e. These calculations are executed subsequent to the embedding process

$$X_{step\_1}=X_{Embedding}+MSR(LayerNorm(X_{Embedding}))$$

In this context, MSR represents a multi-scale retention mechanism akin to the multi-head self-attention mechanism, where the number of multi-heads $h=d_{\mathrm{model}} / d$ is first calculated, $d_{\mathrm{model}}$ denotes the feature dimension, and $d$ is the head dimension. Then it is calculated according to the following equations

$h_{i}=Retention(X_{Embedding},1-2^{-5-arrange(0,h)})$

$H=GN(Contact(h_{1},h_{2},...,h_{i}))$

$$MSR(X_{Embedding})=(swish(X_{Embedding}W_{o})\bigotimes H)W_{o}$$

Retention is a mechanism that integrates recursive and parallel processes. By substituting Softmax with a position-dependent exponential decay term, the computational process is simplified, facilitating the preservation of information from prior steps through decay. Additionally, a complex space is introduced to supplant absolute or relative positional encoding, thereby simplifying the transition to a recursive format. The retention mechanism also incorporates decay rates at various scales, improving the model's capacity to represent complex patterns. It exploits the scale invariance of the GroupNorm technique to boost the numerical accuracy of the Retention layer. Through this mechanism, the trained model is capable of undergoing parallel training while concurrently executing inference, balancing processing performance and modeling efficacy. The final outcome is produced by utilizing the LayerNorm layer and the FeedForward layer

$X_{step\_2}=X_{step\_1}+Gelu(FeedForward(LayerNorm(X_{step\_1})))$

$$X_{feat\_2}=X_{step\_2}$$

Subsequently, the combination of $X_{feat\_1}$ and $X_{feat\_2}$ is executed to compute the final result.

$$outputs=Linear(Relu(Linear(Concat(X_{feat\_1},X_{feat\_2}))))$$

During the model training, the loss function is calculated as

$$\mathcal{L}_{PMLM}=-\frac{1}{N}\sum_{i=1}^{N} \sum_{j=1}^{L_{i}} log(\frac{exp(y_{ij})}{\sum_{k=1}^{num\_classes} exp(y_{ik})})$$

where $N$ represents the total number of sequences in the batch, $L_{i}$ is the length of the *i-*th sequence,$y_{ij}$ denotes the logarithmic probability of the correct category at the position *j* in the *i-*th sequence, and num_classes signifies the total number of categories in the classification task.
